# Treatment of lower cervical spine fracture with ankylosing spondylitis by simple long anterior cervical plate: a retrospective study of 17 cases

**DOI:** 10.3389/fneur.2024.1300597

**Published:** 2024-07-02

**Authors:** Weifu Chen, Yu Yang, Wenjun Pan, Xinhuan Lei, Zhenghua Hong, Hua Luo

**Affiliations:** Department of Orthopedics, Taizhou Hospital of Zhejiang Province Affiliated with Wenzhou Medical University, Taizhou, Zhejiang Province, China

**Keywords:** ankylosing spondylitis, fracture, cervical spinal cord injury, long anterior cervical plate, anterior approach

## Abstract

**Objective:**

Ankylosing spondylitis (AS), an autoimmune disease, often leads to lower cervical spine fractures, with the potential for severe spinal nerve damage even from low-energy injuries. The optimal treatment approach remains debated.

**Methods:**

A retrospective study involved 17 AS patients with lower cervical spine fractures who received anterior cervical fixation. Most presented cervicothoracic or thoracolumbar kyphosis, with 11 exhibiting neurological deficits. Patient characteristics, clinical data, visual analog scale (VAS), complications, and nerve recovery were analyzed.

**Results:**

No postoperative neurological deterioration occurred. All cases experienced complete fusion of fractures during the follow-up period. Preoperative VAS significantly decreased at 3 days and 3 months post-surgery. Of the 11 patients with preoperative neurological deficits, approximately 54.5% showed improvement post-surgery. No complications were reported, such as esophageal fistula, wound infection, or fixation failure.

**Conclusion:**

Anterior internal fixation is a possible treatment for AS-related lower cervical fractures. This approach ensures satisfactory spinal stability and neurological recovery with proper cranial traction and external fixation post-surgery. Our findings demonstrate that this surgical method is safe and effective.

## Introduction

Ankylosing spondylitis (AS) is an autoimmune disease that mainly affects axial bones, such as the spine and the sacroiliac joints, causing stiffness and rigidity of the sacroiliac joints and spine (1). The affected spine in AS is often unable to withstand relatively normal stress compared to a healthy spine because patients with AS often have varying degrees of decreased bone density or osteoporosis and are more likely to develop fragility fractures of the spine in response to violence. According to relevant literature reports (2, 3), the incidence of spinal fracture in AS patients is four times that of ordinary people, with an incidence of 5–15%. Clinical studies have shown that the lower cervical vertebra is the most common fracture site of AS, and the fracture type has its unique characteristics. It is often a three-column fracture across the intervertebral space, similar to the “bamboo” long lever arm fracture, so this fracture is extremely unstable. According to statistics (4–6), about one-third of patients with AS have fatal injuries after cervical spine fractures and even low-energy injuries can cause severe spinal nerve injuries.

The fracture site of the cervical vertebra in AS is most likely at the lower cervical vertebra and the junction of the cervical-thoracic vertebra. This brings many difficulties to the clinical diagnosis and causes delayed or missed diagnosis. Therefore, it is suggested that a three-dimensional CT or MR Examination should be performed when a fracture is suspected in patients (7, 8). Timely and effective treatment of AS with cervical fracture is essential to restore normal neuromotor function and even save lives. At present, there are apparent disputes about the treatment (9–12): traditionally, doctors do not recommend surgery because of the high rate of surgical complications. However, with the update of the treatment concept in recent years, more and more doctors now recommend surgical internal fixation treatment. In addition, the choice of surgical approach is also controversial. Some surgeons recommend simple posterior cervical fixation, while others recommend anterior cervical fixation and fusion (13–15). However, the stability of anterior fixation has been questioned. Although the posterior approach has advantages in decompression and fixation, there are many problems in position placement, intraoperative fracture reduction, accurate nail placement, postoperative incision infection, etc. In contrast, the anterior approach can better avoid the above problems. The difficulty of the operation is relatively simple and fast. Long segmental steel plates can effectively compensate for the shortcomings in cervical stability after the anterior approach.

This study presents the preliminary clinical and imaging results of the anterior long segmental steel plate fixation treatment of AS with lower cervical fracture. It is intended to explore and analyze the injury characteristics of AS with lower cervical fracture through relevant research results, evaluate the efficacy of this technique in treating AS with cervical fracture, analyze and discuss the limitations and deficiencies of this technology.

## Methods

### Study design and patient population

This is a retrospective clinical study conducted through continuous retrospective analysis at Taizhou Enze Medical Center (Group) from June 2010 to December 2022, and it has been approved by the ethics committee under approval number NO. K20220609. In this study, patients who were not diagnosed with AS or patients who had lower cervical spine fractures in the early stage of disease progression but whose spines were still flexible or had no severe cervical kyphosis were excluded. Finally, 17 patients who received anterior long-segment steel plate (German Mediox) surgery for AS combined with lower cervical spine fractures were selected to be included in this study. Among the 17 patients, 11 were accompanied by spinal nerve dysfunction of varying degrees, and surgery was arranged for them as soon as possible after admission. The basic information, the condition of fracture injury, neurological function classification, and other perioperative details of the patients are shown in Table 1.

**Table 1:** Clinical data of 17 patients managed by long anterior cervical plate.

Case No.	Age (Years)	Sex	D-AS (Y)	Mechanism	Level	Classification of fracture	ASIA grade	TBINS	Plate treatment	BL (mL)	HS (D)	Follow-up (Y)
1	54	M	NA	Hyperextension	C6	II	C	6 D	C4-7	50	12	5
2	35	M	10	Hyperextension	C6-7	I	D	2 M	C5-T1	100	34	9
3	45	M	16	Hyperflexion-Hyperextension	C6-7	I	D	4 D	C5-T1	50	13	7
4	48	M	10	Hyperextension	C5-6	I	D	3 D	C4-7	50	34	7
5	49	M	10	Hyperflexion-Hyperextension	C7	IV	E	3 D	C5-T1	50	37	7
6	45	M	20	Hyperextension	C7-T1	I	E	6 D	C6-T2	30	17	6
7	52	M	28	Hyperextension	C5-6	IV	C	2 D	C4-7	50	11	5
8	44	M	NA	Hyperextension	C7	II	E	3 D	C5-T1	40	32	5
9	49	M	20	Hyperextension	C5-6	I	E	6 D	C4-7	300	18	7
10	48	M	30	Hyperextension	C7-T1	I	D	3 D	C6-T2	100	25	4
11	67	F	10	Hyperextension	C6-7	I	C	2 D	C5-T1	50	43	4
12	58	M	10	Hyperextension	C5-6	I	E	3 D	C4-7	50	23	10
13	36	M	18	Hyperextension	C7	II	C	8 D	C5-T2	700	23	10
14	45	M	20	Hyperextension	C6-7	IV	E	6 D	C5-T1	20	23	1
15	34	M	12	Hyperextension	C6	IV	D	4 D	C5-T1	50	11	3
16	71	M	7	Hyperextension	C7	II	D	6 M	C5-T2	30	11	1
17	56	M	10	Hyperextension	C5-6	I	C	1 D	C4-7	100	18	3

M, Male; F, Female; D-AS, Duration of AS progression; NA, Not applicable; TBINS, Time between injury and surgery; OT, Operation time (minutes); BL, Blood loss (mL); HS, Hospital stay (days); D, Day; M, Month; and Y, Year.

### Data collection

The types of injuries seen on CT scans were determined based on the systems: Caron et al. (16) were evaluated and classified by two experienced surgeons above the deputy chief of spine surgery. The injury score of the American Spinal Injury Association (ASIA) was used to evaluate the degree of neurological injury (17). Evaluation of cervical healing status: Cervical spine anteroposterior and lateral X-ray and three-dimensional CT imaging data. Other clinical data include the Visual analog scale (VAS), intraoperative blood loss, length of hospital stay and complications (such as esophageal fistula, wound infection, failure of internal fixation, cerebrospinal fluid leakage, location loss after fracture reduction, further injury of spinal nerve function, respiratory and cardiac arrest or even death).

### Treatment and surgical techniques

Preoperative management: patients were given stay-in-bed and neck brace immobilization immediately after admission. For those who cannot be fixed with a neck brace, use sandbags on both sides of the neck to improve the cervical spine. This group of patients did not receive traction therapy. After admission, patients with spinal cord injury were given symptomatic treatment such as dehydration, detumescence, nerve nutrition and analgesia, and surgery was performed as soon as possible after complete evaluation and exclusion of contraindications.

#### Surgical position

All patients were placed in the position in a fully awake state, supine position, with the head and neck naturally raised, cranial traction arch placed under local anesthesia, appropriate traction and neck flexion under the lateral monitoring of C-arm X-ray machine, to reduce the displaced cervical fracture as far as possible, while the displaced fracture position was kept in place, and the shoulders and head and neck were fixed. At this time, patients’ heads tend to have a higher place, so lower the head and elevate the feet to meet the surgical position and avoid insufficient blood supply to the head during the operation. If necessary, the skull should be kept in 2–4 kg traction, and the position of the broken end of the fracture should be confirmed by fluoroscopy again to prepare for surgical treatment. Then, general anesthesia through nasal or oral tracheal intubation was used with the assistance of a fiber bronchoscope in an awake state.

#### Surgical methods

The anterior edge of the sternocleidomastoid approach was taken. Treatment of intervertebral fracture: Along the gap between the carotid sheath and the internal cervical viscera to the front of the vertebral body, explore and confirm the fracture location, properly stretch the injured intervertebral space, and clean the fracture fragments. If the fracture extends to the intervertebral disk, the disk is meticulously cleaned, followed by iliac bone grafting. Subsequently, a long steel plate is delicately positioned at the front of the neck and secured with screws for stabilization. In cases where the fracture does not affect the intervertebral disk, only a long steel plate is used for fixation. The fractured end of the vertebral body usually does not require treatment and intervention, but if fracture fragments invade the spinal canal, they should be removed and decompressed. Other treatment methods of bone grafting and plate fixation are the same as those of intervertebral space fracture. Neurophysiological monitoring and protection were used throughout the operation.

#### Postoperative management

The skull traction was continued for 2 weeks after surgery, and the patient was allowed to turn over in bed. Then, the head, neck, and chest braces were fixed for 3 months. When the muscle strength of both lower limbs was equal to or greater than grade IV, the patient was allowed to walk on the ground. For patients with spinal cord injury, comprehensive rehabilitation therapy such as hyperbaric oxygen and limb function exercise should be started at an early stage.

### Statistical analysis

The data statistics and analysis were performed using SPSS 25.0. ANOVA was used to compare VAS scores at different time points before and after surgery. And the comparison of ASIA scores before and after surgery was conducted using the Wilcoxon signed-rank test. *p* value<0.05 was identified as a significant difference.

## Results

### Visual analog scale

The average VAS score of patients before surgery was 6.88 ± 1.27 points, and the neck pain symptoms were relieved after surgery. The average VAS score of patients 3 days after surgery was 2.94 ± 0.97 points, significantly lower than the VAS score of patients before surgery, and the difference was statistically significant (*p* < 0.05). The average VAS score of patients 3 months after surgery was 1.47 ± 0.62 points, which was further reduced than the average VAS score of patients 3 days after surgery, and the difference was still statistically significant (*p* < 0.05).

### The American spinal injury association grade

According to ASIA function rating criteria, 17 patients after treatment did not show any postoperative neurological dysfunction deterioration at the last follow-up, and 11 patients with neurological dysfunction before surgery showed improvement of neurological dysfunction in about 54.5% of them (Table 2). Comparison of neurological function between patients before surgery and the last follow-up showed that there was statistical significance (*p* < 0.05).

**Table 2:** ASIA grading for neurological function before operation and at the end of follow-up respectively among 17 patients.

Preoperative ASIA grade	Case lode	ASIA grade at the end of follow-up				
		A	B	C	D	E
A	0	0	0	0	0	0
B	0	0	0	0	0	0
C	5	0	0	2	2	1
D	6	0	0	0	3	3
E	6	0	0	0	0	6

### Fracture healing situation

**Figure 1:**
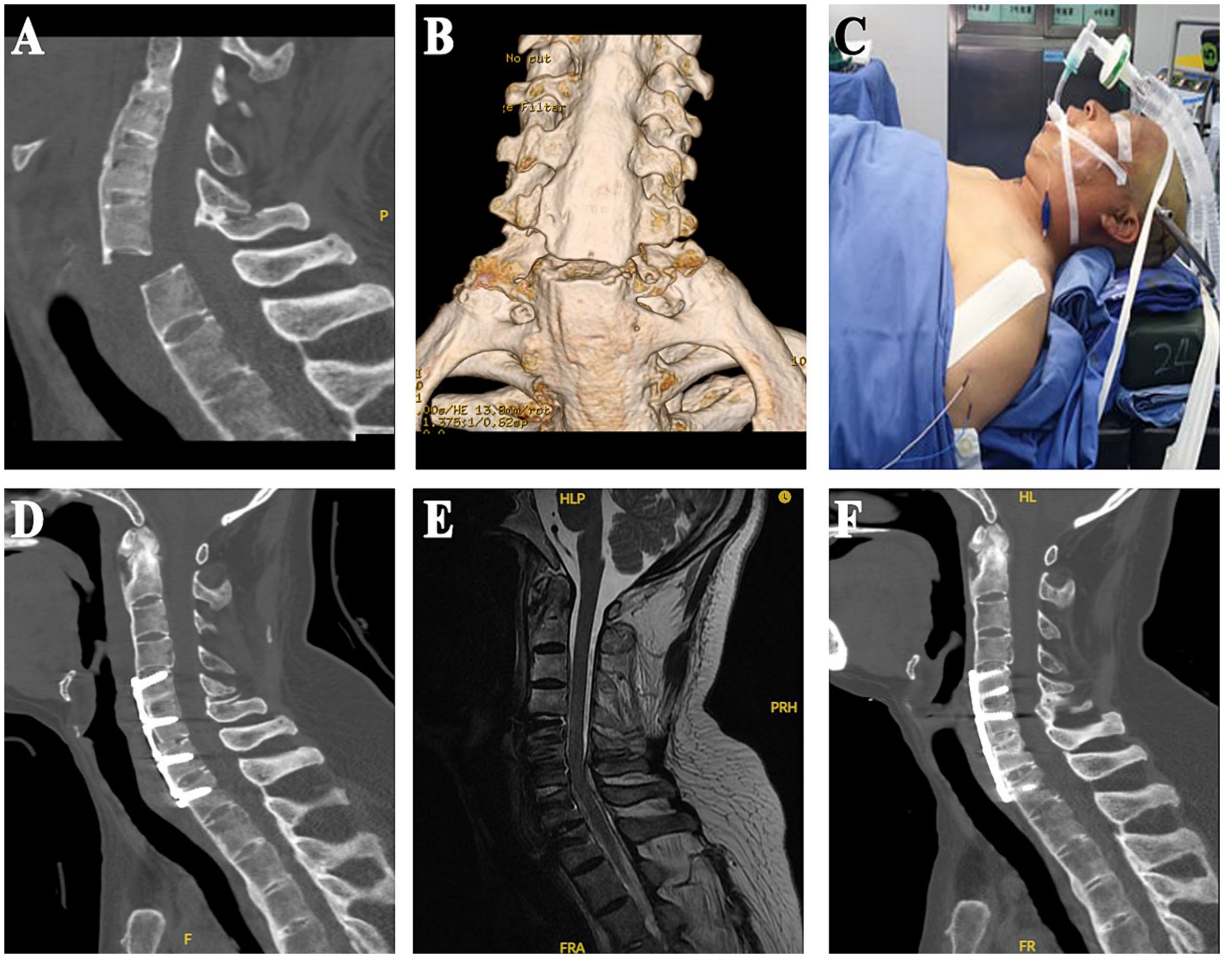
**(A,B)** Preoperative cervical spine CT sagittal and three-dimensional reconstruction images. **(C)** Special postures used by the patient during surgery. **(D,E)** Sagittal CT and MRI images within 1 week after surgery, CT indicated that the internal fixation position was good and stable, and the anatomical morphology of the spinal canal was restored to the maximum extent. **(F)** Sagittal CT images 6 months after surgery.

The postoperative review showed that the internal fixation position was good, and the fracture fixation was firm. All cases exhibited fracture healing during the follow-up period, without occurrences of fracture displacement, non-union, or plate breakage. Typical case was shown in Figure 1.

### Complications

In this group, there were no postoperative complications such as esophageal fistula, wound infection, failure of internal fixation (internal plant rupture, screw rupture, and internal plant loosening, etc.), cerebrospinal fluid leakage, position loss after fracture reduction, respiratory and cardiac arrest, or even death.

## Discussion

Although some earlier literature reported that surgical treatment of these fractures has a high mortality rate, it is clear that surgical treatment has been increasingly recommended (9, 18). Many surgeons recommend posterior surgery, which provides reasonable control and stability at the fracture end by fixing the cervical pedicle (18, 19). However, posterior surgery is also controversial; for example, pedicle nail placement is difficult, dural injury, cerebrospinal fluid leakage, incision infection, and internal fixation looseness are easy to occur, and in patients undergoing posterior surgery, the anterior angle of cervical fracture is not easy to be corrected, resulting in esophageal contusion and esophageal fistula (20, 21). Therefore, a few physicians recommend anterior approach fixation fusion for lower cervical spine fractures in patients with AS. Anterior surgery has the advantages of less trauma, relatively simple operation, thorough decompression, and a high fusion rate. However, the stability of anterior fixation has always been questioned, and it is mostly used in patients without obvious dislocation (2, 22). Internal fixation failure only occurred in simple anterior or posterior internal fixation, so combined anterior and posterior fixation was recommended for such fractures. However, anterior and posterior fixation has higher surgical risks and more complex operations; surgical position placement is also challenging to ensure that it does not cause secondary nerve damage; at the same time, it has all the risks of anterior and posterior surgery because of the trauma and high risk. In anticipation of the heightened risk of esophageal injury during anterior neck surgery for cervical spine fractures in AS, our approach involves a meticulous preoperative assessment, encompassing imaging and clinical evaluation. Proactive measures to address challenges associated with soft tissue swelling include careful surgical planning and anatomical mapping. During surgery, we prioritize optimal exposure, delicate tissue handling, real-time monitoring, and utilize anatomical knowledge to minimize the risk of esophageal injury. This comprehensive strategy aims to enhance patient safety and mitigate potential complications in this challenging surgical context. In this study, we tried to use only the anterior method of cross-segment fixation of the cervical fracture with long segmental steel plates, combined with the process of postoperative skull traction and auxiliary fixation with the head, neck, and chest brace to overcome the shortcomings in the stability of anterior surgery; our research results show that this method has achieved excellent clinical effects in terms of fixation strength, neurological function recovery, surgical complications, and cervical fracture healing.

Our experience is as follows: (1) Before surgery, the patient’s head and neck are positioned high while awake, followed by continuous skull traction under local anesthesia. Dynamic monitoring with fluoroscopy ensures the reduction of cervical fractures and dislocations until satisfactory results are achieved. (2) General anesthesia with endotracheal intubation through the nasal or oral cavity, assisted by a bronchoscope, is preferred. Throughout the procedure, movements of the head and neck are carefully avoided. (3) Comprehensive monitoring and protection of neuroelectrophysiology are maintained throughout the process. (4) Injury to the recurrent laryngeal nerve is minimized through the left neck approach. (5) Given the compression of the anterior column of the fractured vertebra in patients with AS, optimal reduction of the middle and posterior columns is achieved by meticulously removing and scraping the injured intervertebral disk endplate. This is performed without decompression to the posterior edge of the vertebral body, coupled with gentle and appropriate stretching. Autogenous tricortical iliac bone grafting is then implemented. (6) While many AS patients present with cervicothoracic kyphosis, sternotomy or sternoplasty is typically unnecessary for anterior cervical fixation and fusion, as fixation to the T2 vertebra suffices in most cases. Moreover, due to complete fusion of the cervical and thoracic vertebrae in AS patients, adjacent segment instability resulting from adjacent intervertebral disk injury is not a concern. With an adequately long anterior cervical fixation plate, sufficient stability is provided for the patient’s cervical vertebrae. Cervical fractures in AS patients are treated akin to osteoporotic extremity fractures. We have found that using the longest anterior cervical plate (3 or 4 segments, with 4–6 locking screws at both ends) yields excellent fixation results. The screws penetrate the vertebral endplate for multi-cortical and multi-angle fixation, resembling the principle of AO limb internal fixation. Consequently, screw loosening and withdrawal are rare in our cases. Furthermore, to prevent potential internal fixation failure, each patient receives an autogenous iliac bone graft and continuous axial cranial traction protection postoperatively, allowing for axial movement and repositioning.

Regarding the necessity for orthopedic intervention, the consensus among most scholars is that cervical fractures in AS patients present an opportunity for kyphosis correction, as these fractures often involve all three columns and resemble surgical osteotomies (20). We assert that orthosis significantly heightens surgical risks in AS patients with lower cervical fractures, and we do not recommend excessive kyphosis correction via the anterior approach. Instead, following anterior decompression, a modest distraction and robust bone grafting to support the cervical anterior column, along with effective long-segment steel plate fixation, offer a simple, safe, and secure solution.

Regarding the rehabilitation of neurological function, postoperative rehabilitation of lower cervical fracture patients with spinal cord injury is arduous based on AS. Neurological recovery in these patients is often inferior to non-AS patients with lower cervical fractures due to the disease itself. Kouyoumdjian (23) reported that most of the deaths were due to quadriplegia patients with severe spinal cord injury before surgery (grade A or B according to ASIA). We excluded grade A cases from our analysis as there were none present in our study cohort. The operative time of our anterior treatment is relatively short, and early comprehensive treatment such as hyperbaric oxygen within 1 month after surgery is also one of the reasons for the better recovery of patients.

## Limitations

This study also possesses several limitations. Retrospective analysis may introduce recall bias among patients. Additionally, the relatively small sample size and absence of a control group limit the generalizability of the study results. These constraints underscore the necessity for further enhancement through multicenter, prospective randomized controlled clinical trials to validate the findings of this study.

## Conclusion

Anterior cervical long plate surgery is a possible treatment for AS combined with cervical spine fractures due to its simplicity, shorter operation time, and lower bleeding risk. This approach shows high success rates in fracture healing, providing satisfactory spinal stability and neurological function improvement. With the addition of cranial traction and external fixation postoperatively, it proves to be a simple, safe, and effective treatment, especially for surgeons or hospitals unable to master the posterior approach. In addition to surgical segment, the selection of surgical approach should also consider the patient’s bone quality, oedema plane of spinal cord injury, and whether there are Andersson lesions.

## Data availability statement

The original contributions presented in the study are included in the article/supplementary material; further inquiries can be directed to the corresponding authors.

## Ethics statement

The studies involving humans were approved by Enze Hospital of Taizhou Enze Medical Center. The studies were conducted in accordance with the local legislation and institutional requirements. The participants provided their written informed consent to participate in this study.

## Author contributions

WC: Conceptualization, Data curation, Funding acquisition, Investigation, Methodology, Project administration, Resources, Writing – original draft. YY: Project administration, Resources, Data curation, Formal analysis, Funding acquisition, Investigation, Writing – original draft. WP: Methodology, Project administration, Resources, Supervision, Validation, Visualization, Writing – review & editing. XL: Formal analysis, Funding acquisition, Project administration, Resources, Visualization, Writing – review & editing. ZH: Project administration, Writing – review & editing, Investigation, Supervision. HL: Supervision, Writing – review & editing, Formal analysis, Software.
